# The Patient Lived-Experience of Ventral Capsulotomy for Obsessive-Compulsive Disorder: An Interpretive Phenomenological Analysis of Neuroablative Psychiatric Neurosurgery

**DOI:** 10.3389/fnint.2022.802617

**Published:** 2022-02-22

**Authors:** Adriel Barrios-Anderson, Nicole C. R. McLaughlin, Morgan T. Patrick, Richard Marsland, Georg Noren, Wael F. Asaad, Benjamin D. Greenberg, Steven Rasmussen

**Affiliations:** ^1^Warren Alpert Medical School of Brown University, Providence, RI, United States; ^2^Department of Neurosurgery, Warren Alpert Medical School of Brown University, Providence, RI, United States; ^3^Psychiatric Neurosurgery Program, Butler Hospital, Providence, RI, United States; ^4^Department of Psychiatry and Human Behavior, Alpert Medical School of Brown University, Providence, RI, United States; ^5^Department of Neuroscience, Brown University, Providence, RI, United States; ^6^Carney Institute for Brain Science, Brown University, Providence, RI, United States; ^7^Center for Neurorestoration and Neurotechnology (CfNN), Providence VA Medical Center, Providence, RI, United States

**Keywords:** psychiatric neurosurgery, obsessive-compulsive disorder, qualitative study, ventral capsulotomy, lived experience

## Abstract

Ventral Capsulotomy (VC) is a surgical intervention for treatment-resistant Obsessive-Compulsive Disorder (OCD). Despite clinical studies, little is known about patient perception and lived experience after neurosurgery for severe OCD. To examine the lived experiences of patients who have undergone VC for severe, treatment-resistant OCD through qualitative analysis. We conducted semi-structured interviews with six participants treated with VC for OCD. Interviews were analyzed using Interpretive Phenomenological Analysis. The following themes emerged: (1) After years of conventional treatments, patients felt neurosurgery was their “last hope” and described themselves as “desperate,” (2) While some described the surgery as a “supernatural experience,” patients also demonstrated understanding of the scientific procedure, its risks and potential benefits, (3) The surgical experience itself was positive or neutral, which was linked to trust in the clinical team, (4) Post-surgery, participants described months of heightened fear as they awaited lesion formation and functional improvement. (5) Patients consistently contextualized outcome in the context of their own life goals. Patients undergoing VC have positive views of this neurosurgical intervention, but psychiatric neurosurgical teams should anticipate patient discomfort with the time needed to achieve behavioral improvement following surgery and emphasize the importance of post-operative psychiatric care.

## Background

Affecting approximately 2.3% of the United States population, OCD is defined by anxiety-inducing, intrusive thoughts (obsessions) and recurrent compulsive behaviors (compulsions) that manifest as an attempt to reduce obsession-induced anxiety (Rasmussen and Eisen, 1997; Ruscio et al., 2010; Rasmussen et al., 2018). Standard treatment for OCD includes psychopharmacologic and behavioral interventions, but approximately 10% of patients are severely impaired with medically-intractable illness (Greenberg et al., 2003; Shah et al., 2008). Neurosurgical intervention is recognized as an efficacious option for a subset of these patients (Greenberg et al., 2003; McLaughlin et al., 2016; Rasmussen et al., 2018).

Ventral Capsulotomy (VC) has emerged as a successful procedure for patients with medically-refractory OCD. VC involves creating lesions in the brain with various modalities including Gamma Knife Radiosurgery (GKRS) and MRI-guided, Laser Interstitial Thermal Therapy (LITT) in the anterior limb of the internal capsule/ventral striatum (Miguel et al., 2018; Rasmussen et al., 2018). A recent cohort study found that 59% of patients with severe, treatment-resistant OCD treated with gamma knife VC experience a significant reduction in OCD after one year (Lopes et al., 2014; Rasmussen et al., 2018). Numerous studies and a recent meta-analysis have corroborated these findings and also shown that VC improves associated depression, anxiety, functional outcome, and quality of life for patients (Sheehan et al., 2013; Lopes et al., 2014; Brown et al., 2016; Rasmussen et al., 2018; Kumar et al., 2019).

Despite these findings with standard symptom-based outcome measures, no studies to date have explored patients’ perceptions of VC for OCD. Many ways in which patients improve and conceptualize their improvement are not fully captured by standard psychiatric assessments. Patients’ qualitative perspectives on the experience of undergoing psychiatric neurosurgery add an important understudied dimension to outcome and reveal how patient attitudes may impact post-surgical outcomes. This study sought to examine the lived experiences of patients who have undergone VC for severe, treatment-resistant OCD.

## Materials and Methods

### Study Design

In this retrospective qualitative study, we employ Interpretive Phenomenological Analysis (IPA) to examine patient experiences of VC. IPA is a qualitative method developed by Smith et al. (2009) “to capture experientable dialog” (Murphy and Perera-Delcourt, 2014). IPA combines phenomenological, hermeneutic, and idiographic theory to capture participants’ lived experiences in their own terms (Murphy and Perera-Delcourt, 2014). This methodology has been previously employed in both neurosurgical and psychiatric studies (Crist and Tanner, 2003; Murray, 2004; Murray and Harrison, 2004; Smith et al., 2009; Murphy and Perera-Delcourt, 2014; Mulroy et al., 2017).

### Participants and Sampling

Seven interviews were conducted. One participant withdrew from the study after the interview and their data was removed from the study and not included in subsequent analyses. Given the method of in-depth interviews and epistemology of analysis in IPA, sample sizes often range from 6 to 8 participants. Our study sample was recruited by directly contacting all participants for whom we had intact contact information that received VC at our institution. All respondents to our request for an interview were included in the initial research study. The population of individuals who undergo this type of surgery is very small, and our institution represents one of those with the longest track record of neuroablative surgery for OCD. Therefore our research sample represents nearly 10% of the entire capsulotomy sample treated at our program over the past 25 years. We conducted thematic analysis described below until data saturation was achieved in order to ensure that the in-depth studies completed were representative of the features of unique lived-experiences of patients who received GK for OCD. Inclusion criteria for the study included: at least 18 years of age; having undergone VC for OCD previously after having qualified for surgery, and being competent in English. All participants underwent VC at our research program after extensive screening and a detailed informed consent process by a multidisciplinary team of ethicists, neuropsychologists, psychiatrists, neurologists, and neurosurgeons, described in Rasmussen et al. (2018). Participants were aged 28–51 (38 ± 8.5; mean ± standard deviation). Participants either underwent GKRS (*n* = 5) or LITT (*n* = 1) to create bilateral lesions in the ventral portion of the anterior limb of the internal capsule (vALIC) 8–10 mm anterior to the posterior border of the anterior commissure. The time of postsurgical follow-up ranged from 6 months to 24 years (Table 1). Given the small sample size and the detailed nature of the experiences presented here, participants have not been identified by subject ID, surgery type, or gender in the data presentation and results of this investigation as much as possible. All six participants are represented in the illustrative quotes provided.

**TABLE 1:** Research participant information.

Participant	Age	Sex	Surgical intervention	Time since VC	YBOCS at: (Baseline)/(time of interview)*		Surgical outcome
1701	51	M	GKRS	10 months	37	36	Non-responder
1702	36	M	GKRS	1 year	33	25	Non-responder
1703	30	M	GKRS	8 years	39	29	Partial responder
1704	41	M	GKRS	6 months	31	19	Full responder
1705	42	M	GKRS	24 years	40	2	Full responder
1801	28	F	LITT	7 months	33	21	Full responder

*“Time of interview” refers to the YBOCS collected closest to the time of the interview which was within 2 months for four participants and 5–7 years for participants 1703 and 1705, respectively.

### Data Collection

We conducted semi-structured interviews ranging from 30 to 120 mins with each participant. All interviews were conducted by one investigator (ABA) either over the phone (*n* = 4) or face-to-face (*n* = 2). Semi-structured interviews with open-ended questions were utilized to allow participants to guide the discussion of their experiences surrounding surgery and OCD. An interview outline with key questions and topics was developed to aid discussion (Supplementary Material). All interviews began with the broad statement “Tell me about your experience with OCD and surgery for OCD?” and follow-up questions such as “what was that experience like?” and “can you say more about that or explain further?” were used to facilitate the interview process. In keeping with the interview strategy commonly employed in IPA, the flow of the interview was guided by participants, who discussed their experiences in the order they chose; the interviewer used probes from the interview guide to ensure that key experiences were addressed in each interview. The most recent Yale-Brown Obsessive-Compulsive Score (YBOCS) and demographic data including age and sex were collected at the time of the interview. In addition to the qualitative interviews, outcome was assessed for all interviewed participants using the YBOCS. A decrease in YBOCS from baseline of ≥35% was considered a full response, a decrease of ≥25–34% was a partial response, and all others were considered non-responders as previously described (Rasmussen et al., 2018).

### Data Analysis

Participant interviews were audio-recorded and transcribed verbatim. Transcripts were analyzed in detail and annotated by two independent reviewers (AB-A and MP).

Utilizing an IPA approach, we conducted in-depth analysis of each participant’s specific lived-experience of OCD, VC for OCD, and the personal significance and meaning of these experiences (Smith et al., 2009). Transcripts were read multiple times by each reviewer noting phenomena discussed by participants and paying particular attention to descriptions, metaphorical language, and lexical features unique to each interview. Emerging themes were identified independently by both reviewers, and after the initial analysis, both reviewers met to discuss the emerging super-ordinate and subordinate themes identified. Both reviewers discussed the wording and definition of each theme in order to develop a consistent coding scheme. Both reviewers then independently re-reviewed each transcript with the themes in mind to verify them using specific evidence in the transcripts. Reviewers then discussed the findings and agreed upon each assessment of the individual participants and themes to validate the credibility, transparency, and reliability of the data. The findings were presented extensively to all the co-authors in order to discuss inter-rater agreements and disagreements, as well as to leverage the extensive clinical experience of the research co-authors with the larger VC cohort patient population at this institution to gain insights and assess interpretation of participant language.

Reviewers (AB-A and MP) each assessed all transcripts to determine which themes were recurrent across multiple transcripts to identify clustered experiences and themes. After this analysis, a third review was undertaken by NM to ensure the credibility, consistency, and significance of the identified super-ordinate and subordinate themes. All analysis was conducted by hand.

### Research Team Reflexivity

All interviews were conducted by the first author, a male medical student undertaking an MD. He studied IPA with undergraduate advisors in science, technology, and society studies and medical anthropology as a part of the completion of his undergraduate thesis from which this study developed. The study co-authors included members of the psychiatric neurosurgical research program at our institution with a collective experience of more than 30 years in treating and studying the surgical treatment of patients with severe OCD. The first author had no prior relationship with the study participants in the study. Study participants were made aware of the student researcher and his affiliation, but no other information or characteristics about the interviewer were disclosed. The first author maintained a reflective journal of his reactions and thoughts during the study and shared these reflections with the study team throughout the study. The first author’s clinical experience and study of IPA allowed for in-depth interviewing, but he had not previously worked with participants as surgical patients as the other clinical team members had, allowing for a degree of analytic distance. For subsequent analyses, the psychiatric neurosurgical team’s experience with the patient population served to inform and contextualize the emerging data.

## Results

Six participants were interviewed in this study between March 2017 and July 2018 (Table 1). Table 1 contains demographic and surgical outcome data for each participant. All participants underwent GKRS except one who underwent VC using LITT, a critical distinction since GKRS is an awake, non-invasive procedure, and LITT was conducted with general anesthesia.

Transcript analysis revealed four superordinate themes: (1) the *lived experience of treatment-resistant OCD*, (2) the *pre-surgical conceptualization of the operation*, (3) the *experience of the surgery*, and (4) the *post-surgical experience.* Tables 2,3 summarize the identified subordinate themes for each subject. The first super-ordinate theme on *lived experience* included the subordinate themes of “feeling stuck” and “OCD as an intrusive enemy (Table 2).” The theme of *pre-surgical perception* included subordinate themes such as “desperation for surgery,” viewing surgery as a “last hope,” viewing surgery as a magical/supernatural experience, and understanding the scientific rationale for surgery (Table 2). In examining the *experience of surgery* we found that patients had a positive surgical experience, many described the surgery as a means to an end and were unbothered by technical aspects of the surgical experience (Table 3). The *post-surgical experience*, often marked by fear of treatment failure, elucidates how patients assess outcomes with personal metrics, goals, and improvement in their own lives (Table 3).

**TABLE 2:** Subordinate themes: lived experience of Obsessive-Compulsive Disorder (OCD) and pre-surgical conceptualization of lesion procedure.

Participant	Lived experience of OCD		Pre-surgical conceptualization			
	Feeling stuck	Intrusive enemy	Desperation for surgery	Surgery as a “last hope”	Surgery as magical or supernatural	Understanding of science
1701	X	X	X	X	X	X
1702	X	X	X	X	X	X
1703	X					X
1704	X		X	X		
1705	X		X			X
1801	X	X	X			X

**TABLE 3:** Subordinate themes: the experience of surgery and the post-surgical experience.

Participant	Experience of surgery			Post-surgical experience
	Positive surgical experience	Positive view of care team	Means to an end	Fear or worry during the treatment waiting period
1701	X		X	X
1702	X	X	X	X
1703		X	X	
1704	X	X	X	X
1705		X		
1801		X	X	

(1) *The experience of treatment-resistant OCD* was described extensively by each participant who all felt that the many years of debilitating disease was their primary motivator to undergo psychiatric neurosurgery. Many participants described how OCD symptoms contributed to them “feeling stuck” both literally, while completing repetitive compulsions, and in the sense of falling behind in their lives due to hindering illness:

“It’s like I’m a prisoner in my mind.”

“I would spend weekends in bed…I would… take a 3-h shower every day.”

“It was very difficult to get anything done in less than 12 h. And so it took me quite a while to accomplish anything at that point. I think when you are kind of in that situation it is kind of difficult to focus on what’s going on around you. You’re just trying to complete the rituals in a timely fashion so that you can get something done.”

”I just wasn’t really able to communicate with my friends and family, or really keep in touch with people on a regular basis because of the amount of effort it took to get out of bed, get dressed or even get out the door and go visit people. I think I was very socially behind…”

Participants often framed their OCD as “egodystonic,” which means patients characterize the illness as separate from their true feelings or desires and often personify aspects of their individual psyche that they view as an “other,” distinct from how they view their “true” selves (Denys, 2011; Brady, 2014). These individuals often described their OCD as an “intrusive enemy” that constantly plagued them:

“You know I can open the door and go, but it’s [OCD] got a grip on me, it… controls me.”

“It’s like you have two brains. You’ve got your OCD brain and your regular brain, and you can’t shut the OCD brain off… It’s like you have this nasty friend OCD, which you have no choice but to accept that you have it and live your life with it.”

(2) The *pre-surgical conceptualization of the operation* was marked by “desperation” for surgery, a “last hope” for the treatment of OCD. Patients occasionally used magical/supernatural lexicon to describe the surgery, however, nearly all explicitly discussed having a significant understanding of the scientific underpinnings of the operation prior to surgery.

Nearly every participant expressed notable “desperation” to undergo surgery for the relief from OCD symptoms they hoped it would provide:

“I think somebody, was asking my wife … if [I was] worried about the brain surgery and she was like “no, he’d let me do it.” I’d let her do it. If I didn’t wake up, I’d be fine with it too. I would’ve preferred that to just years of OCD.”

“It was so bad after my daughter was born that I was willing to do anything. Even though a brain operation is very serious …I wanted so bad to have the surgery because I knew the relief that [a previous patient] had gotten.”

Desperation was a concordant view amongst nearly all participants and was often accompanied by the expressed view that neurosurgery was a source of “hope” for patients after years of failed interventions:

“If it means moments of peace in your brain you’d be willing to have that. Because my obsessions before the surgery were just constant … I felt hopeless you know. And the surgery was my hope to get better.”

For many, surgery was a source of hope but some presented the perspective that psychiatric neurosurgical intervention was the last available treatment option and thus their “last hope” for treating their OCD symptoms:

“I guess that’s probably what I felt the most nervous about. Because if it didn’t work, then what?”

Patient descriptions of the rationale to undergo VC sometimes included magical or religious language. One participant, for whom religious obsessions were a part of their pathology, described how he came to decide on his surgery: “the feeling I got I believe was from Jesus from Mary to do the surgery and I did.” Despite the presence of magical/religious language, patients consistently based their decisions on scientific rationale and demonstrated detailed knowledge of the surgery, their OCD, and appropriate expectations of VC:

“I thought all the thoughts were my fault, even though they are intrusive and it’s caused by you know the glitch in the brain as you well know… I felt great about it because I knew if there was a chance I could get 1% better, then it’s worth it.”

“I guess based on the information we received about the gamma knife surgery I guess we felt that this was a safer option anyway. Just because it didn’t involve any open surgery or any holes in the skull or anything.”

“I don’t know if you knew all this, but they had started doing the lesions smaller and only doing a single shot. One in each internal capsule… So the only thing I was a little hesitant was that the lesion was going to be smaller. But in turn, since I could not get the double shot, I knew that doing the single shot was worth the risk.”

(3) *The experience of surgery* was unique for each participant who described their experiences and feelings on the day of operation. Overall patients had a generally positive experience of surgery which they attributed to positive interactions with the clinical care team:

“I was happy because we left the hospital. We went straight to the hotel room. We just ate and went to sleep.”

“Oh, I slept through most of it… And the people at Butler [mentions specific clinicians]… They were very approachable… and helpful.”

“Once that was the case they brought me down to the gamma knife center and I was placed in the gamma knife machine. And I believe I was there for about 70 mins for treatment of each side of the brain. And they did a very good job of keeping me comfortable during that time and so I feel that the surgery and the process of undergoing the surgery was very well tolerated… I had a very pleasant experience with both procedures.”

Trust in and explanations from the psychiatric neurosurgical team were also important for patients undergoing this procedure and may have been even more of a consideration in the LITT procedure:

“So I had the [LITT] procedure, and I felt a little nervous because that one hadn’t been done as often as the gamma knife so it felt really experimental and new… the doctors said they can be more precise with the procedure so that made me feel better. Even though they say it’s really precise, I guess it could affect other things and you don’t know what could happen when they’re making a lesion in the brain.”

Many of the participants also expressed the notion that the surgery was a means to an end, contextualizing any discomfort they experienced during surgery as better than “just years of OCD.” For many of the participants, their experience of the specific surgical procedure was generally neutral or positive in part because the operation represented a potential source of relief.

(4) *The post-surgical experience* was discussed extensively by each participant who described significant anxiety in the post-operative “waiting” period before seeing symptom improvement. One participant described this phenomenon as follows: “You may not start healing your OCD for like 6 months and that was depressing.” All patients were interviewed at different times post-operatively and had experienced differing degrees of improvement at the time of the interview (Table 1). Despite differences in outcome at the time of the interview, some participants described anxiety and confusion concerning their improvement in the waiting period after surgery:

“You know I expected to be better than what I am, to be honest with you. At this point, almost a year. And I know… there’s ups and downs… you know I don’t know. I mean the lesions have already formed, they formed at like three months, you know. …I just don’t see how I’m going to get better if they’ve already formed. I don’t understand that.”

Other patients who experienced significant improvement described the same waiting period in a more positive light, particularly if they had continued behavior therapy after surgery:

“After each of the procedures, I guess I didn’t notice a significant difference right away. But I think about a year later I got to a point where I felt like I was much more ready to start doing regular behavioral therapy and participate in the therapy more fully.”

While participants were aware of the YBOCS as an objective metric of improvement, all participants consistently contextualized improvement in their own lives as a measure of their own quality of life, noting specific life goals or experiences with OCD as metrics for success:

“But I had 5 days … [when I] had hours of peace, like hours. Oh my gosh, we’re talking like 3 or 4 h of not even realizing you’re obsessing which is like an absolute miracle for me.”

“Before…It became very difficult to take care of myself. I think you know if left to my own devices I may have starved to death at that point just because I wasn’t able to get out of bed on my own or you know get food or get nourishment on my own… [After the surgery] I was more able to kind of focus on the things that were going on around as opposed to solely focusing on the rituals… I was able to start working with a tutor … and do some preliminary studying and preparing for the high school equivalency exam. I think I was at least able to get to the bathroom and back without help.”

“I’m in school now. School is a little bit easier, it’s still really hard, but it’s easier to do my work and …go to class. I don’t spend as much time worrying about different things. I don’t have to dread showering and stuff like that has gotten easier. So that’s good!”

## Discussion

While qualitative research is a less common method of inquiry in neurosurgery, it has been used to understand patient experiences of various neurosurgical procedures (de Haan et al., 2013, 2015; Mulroy et al., 2017; Shih et al., 2018) IPA is a well-established method ideally suited for in-depth exploration of individual narratives with a small sample of participants (Smith, 1999; Murray, 2004; Murray and Harrison, 2004; Smith et al., 2009; Hammer et al., 2012; Kalhovde et al., 2013; Murphy and Perera-Delcourt, 2014; Toye and Jenkins, 2015; Malterud et al., 2016). To date, there have been few qualitative studies examining the patient experience of psychiatric neurosurgical procedures (de Haan et al., 2013, 2015), and no studies thus far have examined the patient experience of stereotactic lesion procedures for OCD, despite decades of practice (Lopes et al., 2014; Spofford et al., 2014; Miguel et al., 2018; Rasmussen et al., 2018; Kumar et al., 2019).

Our study of severely ill, intractable OCD revealed that patients often feel stuck or limited by the disease as they feel forced to complete compulsive rituals in response to unwanted obsessions. This finding was consistent with other phenomenological studies examining the experience of OCD in both severe and mild cases, suggesting it is a common phenomenon (de Haan et al., 2013; Murphy and Perera-Delcourt, 2014). We also observed that some describe OCD as a separate entity in their mind that plagues them with obsessions of which they have little control. This finding was consistent with previous phenomenological data which supports the diagnostic view that individuals with OCD, an ego-dystonic illness, have tremendous insight about their illness and understand that their behavior is abnormal, excessive, and negatively impacts their well-being (Oulis et al., 2013; Brady, 2014). Taken together these findings suggest remarkable consistency of these specific experiences in the phenomenology and thus clinical appearance of OCD, which is significant given the importance of reliable diagnostic criteria.

These data also reveal unique features of the experience of severe, treatment-resistant OCD in terms of participants’ pre-surgical perspectives. Notably, though perhaps unsurprisingly, nearly all participants expressed having experienced significant “desperation” to undergo VC. This was in part due to frustration many experienced after years of failed medication and behavioral therapy. This view was accompanied, for some, by the expression that surgery was their last hope to see improvement given that in order to qualify for psychiatric neurosurgical procedures patients must have exhausted conventional treatments (Garnaat et al., 2014). The described desperation for surgery may also stem from additional structural barriers patients face to receive psychiatric neurosurgical care such as practitioner attitudes toward referral for surgical evaluation (Cormier et al., 2019). Given the proven efficacy and safety of psychiatric neurosurgical procedures and how few patients undergo such operations, further study exploring possible barriers to surgical evaluation may be warranted (Pepper et al., 2015; Cormier et al., 2019).

Desperation and the sense that surgery is the last option may be themes consistent with elective surgery for intractable illness given similar findings in phenomenological studies on bariatric and refractory epilepsy surgery (de Oliveira et al., 2014; Shih et al., 2018). The perspective of surgery as “a last hope,” however, is at odds with the clinical view that neuroablative procedures are not the last resort but an adjunctive treatment to standard care (Rasmussen et al., 2018; Barrios-Anderson and McLaughlin, 2020). Psychiatric neurosurgical teams should be particularly mindful of these views because patients are often willing to do anything for symptom relief, and conversely, may react poorly if they do not perceive improvement after surgery (Ford, 2009). This uniquely vulnerable position is exemplified by the high risk of suicidal ideation and suicidality in this surgical population before and after VC for severe OCD (Fernández de la Cruz et al., 2017; Rück et al., 2017, 2008), a risk that may be of particular concern in patients with minimal to no improvement after surgery. Clinicians should consider carefully addressing the perception that surgery may be the “last hope” for patients during the informed consent process by providing insight about options and strategies that exist after surgery for this patient population.

Some patients used religious or supernatural concepts when making decisions about surgery, yet nearly all understood the scientific details of VC surgery. Comorbid psychiatric illness and personality disorders are relatively common in cases of severe, treatment-resistant OCD (Pallanti and Quercioli, 2006; Pinto et al., 2006; Rasmussen et al., 2018), and our data suggest that descriptions of surgery with magical or religious language should not be taken as a sign that patients misunderstand the scientific basis of surgery.

Overall VC was well-tolerated by patients who underwent stereotactic lesions with either GKRS or MRI-guided LITT who attributed their positive experience of surgery to their trust and appreciation of the clinical team.

After surgery, patients described the waiting period to see if they would improve, and some described fear, anxiety, and depression at the idea of not improving during that waiting period, which published data suggests can last several months (Brown et al., 2016; Miguel et al., 2018; Rasmussen et al., 2018). This is an important consideration in the post-operative management of this patient population especially given the high rates of co-morbid depression and anxiety (Pinto et al., 2006; Rasmussen et al., 2018). Providing safeguards such as close psychiatric follow-up in the postoperative period is essential as the risk of worsening comorbid illness or self-harm may be increased, either due to perception that their OCD is not improving, or that their overall social functioning has not improved, even if there is an improvement in OC symptomatology. Once patients did experience improvement they described success in the context of their individual lives, or “fields of affordances” as is similarly described in OCD patients undergoing deep-brain stimulation (de Haan et al., 2013). Patients contextualized their outcome based on how surgery allowed them to achieve personal goals, which looked different for different participants. This is notable because while a reduction in YBOCS is a validated metric for symptom improvement, a positive outcome of surgery for some may not necessarily always manifest as a significant reduction in YBOCS. Further study may be necessary to develop validated metrics that better assess individual improvement in the context of their own lives. In this population with severe disease, patients expressed that gaining a few hours of obsession-free thought or mild symptom reduction can make a world of difference.

One limitation of this study is that postoperative interviews were conducted with each participant at highly varying times after their operation. This may have contributed differing degrees of hindsight bias and may not capture valuable pre-surgical patient perspectives and phenomena. Varying interview length and methods of first-person interviews (phone vs face-to-face) may have impacted the qualitative data obtained in unanticipated ways, but the inter-rater consistency in thematic analyses suggests that many significant themes of the patient-lived experience applied to all participants. Further study examining the role of pre-surgical perspectives and attitudes on surgical outcomes for psychiatric neurosurgical procedures is needed. Another significant limitation of this study is that there was only one female participant in this qualitative sample which likely skews the data toward male phenomenological experiences of neurosurgery for OCD and limited our ability to assess experiences or perspectives that may be influenced by gender identity.

## Conclusion

Severe, treatment-resistant OCD is marked by a sense of being trapped in habitual thoughts or behaviors that impede patients’ efforts to lead healthy lives. Patients are often uniquely desperate for surgery, with some viewing VC as the final “hope” for improvement. Clinicians should make every effort to caution patients about surgical risks and highlight the importance of postoperative therapy. Overall patients were satisfied with VC either through GKRS or LITT and noted the positive impact of trust and positive interactions with the psychiatric neurosurgical care team. This further underscores the importance of an expert, multi-disciplinary team in the screening process, as well as for long-term post-surgical care. After surgery, patients experienced anxiety about whether the operation was successful, citing personal experiences as metrics for improvement. As functional neurosurgery continues to evolve as a treatment modality for psychiatric indications, phenomenological illnesses for which biomarkers are only beginning to emerge, qualitative inquiry may prove invaluable as a high-resolution and patient-specific outcome tool.

## Data Availability Statement

Raw data includes identifiable patient information and full transcripts which will not be made available. All secondary analysis and interview study tools are made available with this manuscript.

## Ethics Statement

The studies involving human participants were reviewed and approved by Butler Hospital Institutional Review Board. The patients/participants provided their written informed consent to participate in this study.

## Author Contributions

AB-A designed the study, conducted, and transcribed patient interviews, completed the thematic analysis, and prepared the manuscript. NM supervised this project, designed this study, assisted with analysis, and revised this manuscript. MP assisted with thematic analysis, interpretation of the raw transcript data, and edited the manuscript. RM, GN, WA, BG, SR, and all revised the manuscript, provided expert guidance on study design, contributed to the article, and approved the submitted version.

## Author Disclaimer

The content is solely the responsibility of the authors and does not necessarily represent the official views of the National Institutes of Health.

## Conflict of Interest

The authors declare that the research was conducted in the absence of any commercial or financial relationships that could be construed as a potential conflict of interest.

## Publisher’s Note

All claims expressed in this article are solely those of the authors and do not necessarily represent those of their affiliated organizations, or those of the publisher, the editors and the reviewers. Any product that may be evaluated in this article, or claim that may be made by its manufacturer, is not guaranteed or endorsed by the publisher.

## Abbreviations

VC: ventral capsulotomy
OCD: obsessive compulsive disorder
GKRS: Gama-knife radiosurgery
LITT: laser interstitial thermal therapy
IPA: interpretive phenomenological analysis
vALIC: ventral anterior limb of the internal capsule
YBOCS: Yale-Brown Obsessive Compulsive Score.
