# Supplementary material for: The Patient Lived-Experience of Ventral Capsulotomy for Obsessive-Compulsive Disorder: An Interpretive Phenomenological Analysis of Neuroablative Psychiatric Neurosurgery
Source: Front Integr Neurosci. 2022 Feb 22;16:802617. doi: 10.3389/fnint.2022.802617 (PMC8902594; doi:10.3389/fnint.2022.802617)
Supplement: Supplementary file 1 [file Data_Sheet_1.docx]

**Patient interview outline**

Appendix: Patients Interview Outline and Script

[The following main topics (I, II, III) will be addressed in the interview. They may not all be discussed in the order presented here. The interview will follow the lead of the patient with the following questions only representing potential example questions or prompts that participants may be asked.]

1. OCD experience
   1. Tell me about your experience with obsessive compulsive disorder
   2. When did you first notice symptoms and what were they?
2. Surgical Procedure
   1. How did you come to decide on having surgery to treat your OCD?
   2. What did you think of the surgery?
   3. What was the process of becoming part of the study like?
   4. What type of procedure did you have completed? How did you decide?
   5. What was your feeling towards having an electrode placed in your brain? What was your feeling toward a lesion being made in your brain?
   6. What were the biggest deciding factors when having surgery?
   7. Did any concerns come up? If so, how were they addressed
3. Post-Surgical experience
   1. Describe your journey after the surgery
   2. Did you see, feel, think differently after the operation?
   3. How did the surgery affect your symptoms?
   4. How did the surgery affect your daily life, family, friends?
   5. In hindsight, would you have the surgical procedure completed again?
   6. What was most important for you after the surgery?
   7. Do you think about the surgery regularly now?
